# Mometasone Furoate–Induced Iatrogenic Cushing's Syndrome and Secondary Adrenal Insufficiency: A Case Report

**DOI:** 10.1155/crpe/6109378

**Published:** 2025-06-16

**Authors:** Anna Insalaco, Sara Vandelli, Simona F. Madeo, Patrizia Bruzzi, Viola Trevisani, Barbara Predieri, Laura Lucaccioni, Lorenzo Iughetti

**Affiliations:** ^1^Department of Medical and Surgical Sciences for Mothers, Children and Adults, Post-Graduate School of Pediatrics, University of Modena and Reggio Emilia, Modena, Italy; ^2^Department of Medical and Surgical Sciences for Mothers, Children and Adults, Pediatric Unit, University of Modena and Reggio Emilia, Modena, Italy

**Keywords:** adrenal insufficiency, cortisol, Cushing's syndrome, hypothalamic–pituitary–adrenal axis, intranasal corticosteroids, side effects

## Abstract

Intranasal corticosteroids (INCS) are widely used to treat allergic rhinitis and nasal obstruction. While their safety profile is generally well established, both local and systemic side effects can occur. While it is well-known that a chronic exposure to systemic glucocorticoid treatment could determine Cushing's syndrome (CS) and suppression of the hypothalamic–pituitary–adrenal (HPA) axis, there is less awareness when the administration is topical or intranasal. We report the case of an 8-year-old Caucasian girl who developed Cushingoid features following prolonged INCS treatment—initially with betamethasone and subsequently with mometasone furoate. Endocrine testing revealed undetectable baseline and after stimulation cortisol levels, suggesting a condition of adrenal insufficiency secondary to the prolonged glucocorticoid exogenous administration. Temporary hydrocortisone replacement therapy was required. Even if extremely rare, pediatricians should be aware that high-dose and long-term nasal steroid administration may cause iatrogenic CS, as well as systemic glucocorticoid treatment. Clinical features are characterized by the complications of glucocorticoid excess and by the potential life-threatening complications of adrenal insufficiency. Pediatric follow-up should be scheduled during the prolonged steroid treatment and at discontinuation, with prompt referral to a Pediatric Endocrinologist if signs and symptoms of CS (or adrenal insufficiency) are noticed.

**Summary**

• High-dose and long-term nasal steroid administration can lead to iatrogenic Cushing's syndrome similar to long-term systemic steroid therapy.
• Even if rare, cases of Cushing's syndrome and suppression of hypothalamic–pituitary–adrenal axis have been reported also in otherwise healthy children treated with intranasal corticosteroids only.
• In case of chronic topical glucocorticoid use, clinical practitioners should inform parents about the proper way of administration and potential side effects. Moreover, close pediatric clinical follow-up is required for early detection of side effects.

## 1. Introduction

Chronic exposure to exogenous glucocorticoids (GCs)—especially at high doses or for prolonged periods—is the most common cause of Cushing's syndrome (CS) in children [1].

In most cases, the onset of CS is insidious, resulting in late or under-reported diagnosis. Even if growth failure and excessive weight gain are the most frequent presenting symptoms, CS has a multisystemic involvement characterized by dermatological, cardiovascular, neurological, and psychological symptoms [2].

Fat redistribution can occur with “moon face,” truncal obesity, buffalo hump, and supratemporal and supraclavicolar fat pads [2].

Virilization with apparent precocious puberty may occur, while pubertal delay or menstrual irregularities are also possible in girls due to GC-induced hypogonadism. Moreover, the chronic and prolonged exposure to GCs could result in osteopenia or osteoporosis, increasing the risk of spontaneous fractures. Finally, neurological symptoms such as headaches, mood changes (including emotional lability, irritability, or depression), and sleep disturbances may also be present, even if more frequently observed in adults than in children [1].

Chronic exposure to synthetic GCs might lead to suppression of the HPA axis. The interaction between derivative GCs and the GC receptor, depending on the molecular power, exerts negative feedback on the corticotropin-releasing hormone, producing neurons and pituitary corticotroph cells. This leads to reduced adrenal cortisol production and eventually to adrenal cortical hypoplasia and atrophy. After GCs treatment discontinuation, adrenal insufficiency may occur [3].

We report the case of a girl with iatrogenic CS and secondary adrenal insufficiency following long-term treatment with Intranasal corticosteroids (INCS) for chronic nasal obstruction. This case is noteworthy due to the rare occurrence of iatrogenic CS and subsequent secondary adrenal insufficiency resulting from INCS use, which is generally considered to have minimal systemic absorption. Reporting such cases is crucial to increase awareness among clinicians regarding potential systemic effects even with topical formulations.

## 2. Case Presentation

An 8-year-old Caucasian girl was referred to our pediatric emergency department for asthenia, rapid weight gain (+15 kg in 3 months) with concomitant decrease in growth rate, and hirsutism of recent onset. She also reported recurrent headaches without visual disturbances.

Four months before, the patient was diagnosed with chronic nasal obstruction and obstructive sleep apnea syndrome (OSAS). Since then, therapy with high doses of INCS was started: for the first month with betamethasone spray (six to eight applications/day, approximately 350 μg/day), then with high doses of mometasone furoate spray (at least six applications/day, corresponding to 300 μg/day), which she was still using at the time of evaluation.

**Figure 1:**
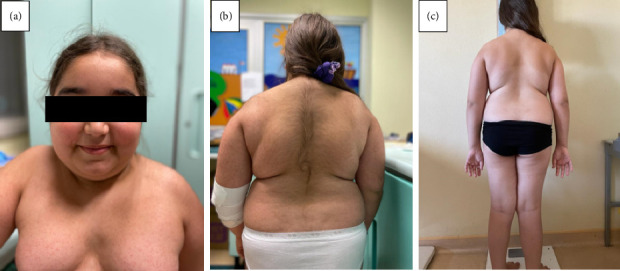
Patient at first examination: (a) front, (b) back, (c) and after 1 year with regression of the phenotypic characteristics.

At the clinical evaluation, she presented Cushingoid features with “moon face,” truncal obesity with abdomen and thigh stretch-marks, facial and truncal hirsutism (Ferriman–Gallwey score = 21), and cervical and axillary acanthosis (Figures 1(a) and 1(b)). Her heart rate was 108 bpm and blood pressure 137/77 mmHg (at the 97° centile for age and height).

**Figure 2:**
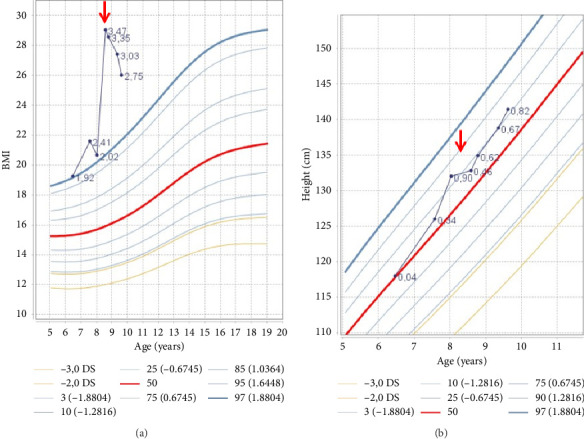
WHO growth charts: (a) BMI curve showing the rapid increase in weight before presentation (pointed with the arrow) and its successful reduction after treatment with hydrocortisone. (b) Height curve showing the decrease in heightening (pointed with the arrow) and its successful increase after treatment with hydrocortisone.

Auxological evaluation confirmed the presence of obesity (body mass index 29.03 kg/m^2^, 3.09 SDS) and a severe growth retardation (velocity growth 1.45 cm/year) (Figures 2(a) and 2(b)).

Laboratory findings showed a normal white blood cell count and renal function with a slight increase in transaminases. Alterations on glucose and lipid metabolism were found, with hypercholesterolemia, hypertriglyceridemia, and insulin-resistance (total cholesterol 286 mg/dL [> 95°p], LDL cholesterol 186 mg/dL [> 95°p], triglycerides 172 mg/dL [> 95°p], basal insulin 17 µUI/mL, glycemia 81 mg/dL, HOMA index 3.4). Severe vitamin D deficiency with secondary hyperparathyroidism was also noticed (Table 1).

Endocrinological work-up (Table 1) revealed low serum concentrations of morning cortisol and ACTH, with undetectable levels of 17-OH progesterone and androstenedione. Renin and aldosterone levels were normal. A 24-h urine collection showed undetectable levels of urinary free cortisol.

A low dose ACTH test was performed (1 mcg Synachten), showing an inadequate secretion of cortisol poststimulus (cortisol peak at 40 min: 1.8 μg/dL; normal range 6.7–22.6 μg/dL), confirming secondary adrenal insufficiency. Cerebral magnetic resonance imaging (MRI) showed neither parenchymal nor pituitary signal alterations, while abdominal ultrasound and MRI showed mild hepatomegaly with steatosis, with no adrenal abnormalities.

Based on her clinical history, physical findings, and biochemical results, a diagnosis of iatrogenic CS with secondary adrenal insufficiency was established. Hydrocortisone replacement therapy (9 mg/m^2^/day in three divided doses) and vitamin D supplementation were started.

Hydrocortisone was gradually tapered, both to prevent adrenal crisis and to avoid recurrence of nasal obstruction. The patient's adrenal function progressively recovered and hydrocortisone was suspended after 5 months, with no relapse (Table 1).

At 1-year follow-up, the patient showed complete clinical recovery, with resolution of hypercorticism signs and improvement of auxological parameters (Table 2, Figures 1(c), 2(a), 2(b)). Furthermore, glucose and lipidic metabolism gradually improved until normalization (basal insulin 7.4 µUI/mL, glycemia 81 mg/dL, HOMA index 1.48, total cholesterol 155 mg/dL, LDL cholesterol 85 mg/dL, and triglycerides 128 mg/dL).

## 3. Discussion

The use of INCS is common in the treatment of allergic rhinitis and nasal obstruction in children. When used alone and at adequate dosage, INCS rarely cause systemic side effects. Absorption of corticosteroids through the nasal mucosa is limited, but a small amount of the dose could be swallowed and absorbed via the gastrointestinal tract, potentially contributing to systemic effects [4].

Even if all INCS are generally considered safe and effective, differences in potency, molecular structure, and pharmacokinetics properties could result in different clinical effects. The risk of systemic adverse effects generally diminishes as the topical potency increases and the bioavailability decreases. Therefore, INCS with a greater glucocorticoid receptor (GR) binding affinity, which is strongly related to the molecule lipophilicity, increases nasal tissue retention and topical potency, reducing the risk of systemic side effects [5, 6].

Moreover, both treatment duration and individual susceptibility are key elements in determining the occurrence and severity of complications during INCS therapy. The association of INCS with inhalator or systemic GC treatment might increase the risk of adverse effects, as the concomitant use of drugs increasing the bioavailability of GC (e.g., AIDS patients on ritonavir, a potent inhibitor of hepatic cytochrome P450, who were also simultaneously taking inhaled steroids such as fluticasone) [7].

Despite their minor impact on the HPA axis compared to systemic GC administration, cases of CS and HPA axis suppression have been reported even in otherwise healthy children treated with INCS alone [8, 9].

Indeed, the overall risk of GC-induced adrenal insufficiency, observed in both children and adults, is estimated at 4.2% with intranasal administration, notably lower than the 48.7% risk associated with oral administration [4]. Most cases of iatrogenic CS following INCS treatment are due to the administration of nasal steroid drops, instead of sprays, which are less likely to be absorbed due to the lower risk of ingestion [8].

Perry et al. described nine cases of children who developed CS following the use of various steroid nasal drops (betamethasone, beclomethasone, fluticasone, and flunisolide). Other authors have reported similar complications associated with the use of dexamethasone nasal drops. In most cases, clinical presentations included facial puffiness, rapid weight gain, growth failure, and hypertrichosis. All patients had been exposed to supratherapeutic doses for at least 4 weeks [8–10].

To the best of our knowledge, no cases of CS have been reported in patients treated with mometasone furoate, which does not seem to suppress the HPA axis when administered at clinically recommended doses (100–200 μg/day) [6]. As reported in Table 3, mometasone furoate, along with other second-generation INCS molecules such as fluticasone propionate and furoate, has high GR binding affinity and lipophilicity, with systemic bioavailability below 1%, which is significantly lower when compared to first generation molecules, such as dexamethasone, budesonide, and beclomethasone diproponiate. These pharmacological features make mometasone furoate one of the preferred INCS options, offering an optimal balance between efficacy and safety. However, given its high potency, it is mandatory to adhere to recommended doses to ensure negligible systemic concentrations and prevent adverse effects [5].

Moreover, interindividual variability also plays a significant role in the risk of adrenal suppression, which may explain why isolated patients exhibit changes in HPA-axis function. The different sensitivity to GC seems to be explained by the discovery that there are multiple GR isoforms generated by alternative splicing. While loss-of-function GR polymorphisms are associated with GC resistance, gain-of-function polymorphisms can increase GC sensitivity [7].

The most appropriate test to confirm the suspect of adrenal insufficiency is a low-dose ACTH stimulation test. To assess the HPA axis recovery, morning serum cortisol, ACTH, and eventually an ACTH-stimulation test should be performed after 24 h of therapy [3].

If complete adrenal suppression is confirmed, hydrocortisone replacement therapy should be initiated at a substitutive dose (8–10 mg/m^2^ per day), followed by gradual tapering. Families must be advised to double the dose in case of acute illness, trauma, or surgical procedures [3]. The recovery time of HPA axis after suppression is highly variable and has not specifically been evaluated in patients treated with INCS. In patients treated with topical GCs, HPA axis recovery time is reported to range from 1 month to 1 year (3.49 ± 2.92 months) [11].

Our patient required GC replacement for 5 months with gradual tapering, both to prevent adrenal insufficiency due to HPA suppression and to avoid recurrence of nasal symptoms.

## 4. Conclusion

In conclusion, we reported the case of a child with chronic nasal obstruction who developed CS following prolonged INCS therapy.

Even if rare when used as monotherapy, prolonged high-dose INCS treatment can lead to iatrogenic CS, with associated complications due to GC excess and potentially life-threatening adrenal insufficiency. INCS should be prescribed at the lowest effective dose and for the shortest time, preferably avoiding superpotent formulations.

It is also important to inform patients and caregivers about the correct way of administration of steroid-containing treatments (modality, quantity, and duration) and the possible side effects.

In case of chronic GC exposure, even with topical therapy, gradual tapering and close clinical follow-up are essential. Prompt referral to a Pediatric Endocrinologist is recommended if signs and symptoms of CS (or adrenal insufficiency) appeared.

## Figures and Tables

**Table 1:** Biochemical and hormonal levels' timeline during the first year of follow-up.

	Time 0	At 2 months	At 5 months	At 9 months	At 1 year	Reference range
25-OH vitamin D (ng/mL)	6.3	26.9	33.9	30.9	19.2	20–100
PTH (pg/mL)	100.0	83.3	87.0	50.6	68.9	15–88
Ca^2+^ (mg/dL)	10.0	9.8	10.1	9.3	9.7	8.5–10.5
Phosphorus (mg/dL)	3.5	5.1	4.9	4.9	4.5	4.0–7.0
ALP (U/L)	80	218	210	195	237	140–400
Cortisol (μg/dL)	0.4	7.7	9.5	9.3	5.2	6.7–22.6
ACTH (pg/mL)	5.1	28.6	22.1	16.8	17.7	4.3–52
Androstenedione (ng/mL)	< 0.240	0.82	0.98	0.49	1.12	0.4–3.4
17-OHP (ng/mL)	< 0.1	0.9	1.1	0.8	0.7	0.2–1.3
DHEAS (μg/mL)	0.03	0.17	0.29	0.41	0.52	0–0.94
Treatment	Hydrocortisone started at 9 mg/m^2^, then progressively tapered		Stop hydrocortisone	No therapy	No therapy	

*Note:* 25-OH-Vitamin D, calcifediol; PTH, intact parathyroid hormone; Ca^2+^, calcium; ALP, alkaline phosphatase.

**Table 2:** Anthropometrics during the first year of follow-up.

	Time 0	At 2 months	At 5 months	At 9 months	At 1 year
Age (years)	8.58	8.78	8.93	9.35	9.62
Weight (kg) (SDS)	51.2 (3.26)	52.0 (3.21)	50.9 (2.96)	52.8 (2.92)	52.0 (2.69)
Height (cm) (SDS)	132.8 (0.46)	134.9 (0.62)	135.3 (0.42)	138.8 (0.67)	141.4 (0.82)
BMI (kg/m^2^) (SDS)	29.03 (3.47)	28.57 (3.35)	27.8 (3.20)	27.41 (3.03)	26.01 (2.75)
Body surface area (m^2^)	1.37	1.40	1.38	1.43	1.43
Blood pressure (mmHg)	137/77	117/60	119/67	126/67	126/67

**Table 3:** INCS pharmacological and pharmacokinetic characteristics (modified by Daley-Yates et al. [5]).

INCS	Receptor binding affinity (relative to dexamethasone = 100)	Lipophilicity^a^	Bioavailability^b^ (%)	Systemic clearance (L/h)
Dexamethasone	100	1.68	75	17
Budesonide	935	2.32	31	84
Beclomethasone diproprionate	1345	3.27	44	120
Flunisolide	190	1.36	50	58
Mometasone furoate	2100	4.73	0.46	54
Fluticasone furoate	2989	4.17	0.50	65
Fluticasone proprionate	1775	3.89	0.51	69

^a^Lipophilicity defined as log10 of the octanol/water partition coefficient.

^b^Bioavailability via nasal route determined in healthy subjects.

## Data Availability

The data that support the findings of this study are available on request from the corresponding author. The data are not publicly available due to privacy or ethical restrictions.
